# Preparing the Home and Community Based Workforce for Remote Monitoring Technology: Lessons From a Qualitative Study

**DOI:** 10.1093/geroni/igaf122.2210

**Published:** 2025-12-31

**Authors:** Erica Perryman, Kathleen Buckwalter, Emily Jones, Jennifer Heck, Diana Sturdevant, Frances Wen

**Affiliations:** The University of Oklahoma Health Sciences Center, Tulsa, Oklahoma, United States; University of Iowa, Iowa City, Iowa, United States; The University of Oklahoma Health Sciences Center, Oklahoma City, Oklahoma, United States; The University of Oklahoma Health Sciences Center, Oklahoma City, Oklahoma, United States; The University of Oklahoma Health Sciences Center, Oklahoma City, Oklahoma, United States; The University of Oklahoma, Norman, Oklahoma, United States

## Abstract

The use of remote monitoring technology for care coordination among community-dwelling older adults (age ≥ 65) increased during the COVID-19 pandemic and continues today. Remote patient monitoring is valuable for older patients because it eliminates barriers to healthcare access and care coordination, better allowing them to age in place. Yet, many professionals in healthcare believe that their older patients are unwilling or unable to use remote patient monitoring technology. Healthcare professionals are challenged to overcome ageist beliefs that contribute to the digital divide among older adults. Likewise, health systems are challenged to develop new approaches to care delivery. Remote patient monitoring offers a new approach to home and community-based services care coordination, but its implementation is less understood. A qualitative descriptive study using multi-perspective interviewing (N = 34, resulting in 7 triads) was conducted to better understand the perspectives of remote monitoring technology among older adults (n = 15), their informal caregivers (n = 8), and their home and community-based service providers (n = 11). Synchrony was found among the three groups about the benefit of remote patient monitoring for the older adult’s care coordination, yet ageist beliefs most often prevented the HCBS provider from introducing remote monitoring technologies to their older adult clients. Lessons from this study inform the workforce development efforts intended to prepare home and community-based service providers for remote patient monitoring as a new approach to care coordination and delivery.

